# Varicella-Zoster Virus IE4 Protein Interacts with SR Proteins and Exports mRNAs through the TAP/NXF1 Pathway

**DOI:** 10.1371/journal.pone.0007882

**Published:** 2009-11-18

**Authors:** Isabelle Ote, Marielle Lebrun, Patricia Vandevenne, Sébastien Bontems, Cahora Medina-Palazon, Evelyne Manet, Jacques Piette, Catherine Sadzot-Delvaux

**Affiliations:** 1 Laboratory of Virology and Immunology, GIGA-R, University of Liege (ULg), Liège, Belgium; 2 Laboratoire de Virologie Humaine, INSERM U758, ENS-Lyon, Lyon, France; Centre de Regulació Genòmica, Spain

## Abstract

Available data suggest that the Varicella-Zoster virus (VZV) IE4 protein acts as an important regulator on VZV and cellular genes expression and could exert its functions at post-transcriptional level. However, the molecular mechanisms supported by this protein are not yet fully characterized. In the present study, we have attempted to clarify this IE4-mediated gene regulation and identify some cellular partners of IE4. By yeast two-hybrid and immunoprecipitation analysis, we showed that IE4 interacts with three shuttling SR proteins, namely ASF/SF2, 9G8 and SRp20. We positioned the binding domain in the IE4 RbRc region and we showed that these interactions are not bridged by RNA. We demonstrated also that IE4 strongly interacts with the main SR protein kinase, SRPK1, and is phosphorylated in *in vitro* kinase assay on residue Ser-136 contained in the Rb domain. By Northwestern analysis, we showed that IE4 is able to bind RNA through its arginine-rich region and in immunoprecipitation experiments the presence of RNA stabilizes complexes containing IE4 and the cellular export factors TAP/NXF1 and Aly/REF since the interactions are RNase-sensitive. Finally, we determined that IE4 influences the export of reporter mRNAs and clearly showed, by TAP/NXF1 knockdown, that VZV infection requires the TAP/NXF1 export pathway to express some viral transcripts. We thus highlighted a new example of viral mRNA export factor and proposed a model of IE4-mediated viral mRNAs export.

## Introduction

In eukaryotic cells, export of mRNAs from the nucleus into the cytoplasm is a complex and well regulated process. In metazoans, mature mRNPs are transported by the essential mRNA export receptor TAP/NXF1 that shuttles between the nucleus and the cytoplasm and escorts competent mRNPs out of the nucleus through direct interactions with nucleoporins lining the nuclear pore [1]. Because of its low affinity for binding mRNAs, TAP/NXF1 needs export adaptor proteins to interface with mature transcripts that are ready for export. So far, the best-characterized adaptor of TAP/NXF1 is the Aly/REF protein [2]. For its recruitment to mRNAs, Aly/REF requires the essential mRNA export factor UAP56 [3] and these two proteins were originally found to be associated with the exon junction complex (EJC) formed during late stage of pre-mRNA splicing [4]. More recent studies have shown that UAP56 and Aly/REF are part of the multi-protein TREX (transcription-export) complex, which is recruited co-transcriptionally to the 5′ end of mRNAs via the cap-binding protein Cbp80 [5] and is essential for the export of both spliced and intronless mRNAs [6], [7]. While UAP56 was shown to be essential for mRNA export in both Drosophila and *C. elegans*, Aly/REF seems to be dispensable, suggesting the existence of additional mRNA export adaptors [8], [9]. Moreover, it has been speculated that the shuttling SR proteins SRp20, 9G8 and ASF/SF2, retained on mRNAs, might also generate export-competent mRNPs. Interestingly, shuttling SR proteins have been shown to promote export of both intronless [10] and intron-containing [11] mRNAs. Thus, these proteins may be export adaptors shared by different mRNA classes. This hypothesis is supported by the fact that, like the adaptor Aly/REF, shuttling SR proteins can directly interact with TAP/NXF1 and can recruit this export receptor to bound mRNAs [12].

In case of a viral infection, in addition to cellular mRNAs, amounts of viral mRNAs have to be efficiently transported to the cytoplasm for translation. For this, several viruses use a similar strategy that involves specific *cis*-acting RNA elements within the intronless transcripts. Among the herpesviral genes, only one *cis*-acting RNA element for mRNA export has been actually described [13]. Instead, it is now established that herpesviruses encode a conserved gene family whose proteins act as viral mRNA export factors that mediate nucleocytoplasmic transport of viral transcripts [14]. This conserved family of proteins contains the ICP27 protein of herpes simplex virus type 1 (HSV-1), the UL69 protein of human cytomegalovirus (HCMV), and the EB2 protein of Epstein-Barr virus (EBV), respectively alpha-, beta- and gamma-herpesviruses. The principal characteristics of these viral mRNA export factors are a nucleocytoplasmic shuttling activity, an RNA-binding domain and the capacity to interact with cellular mRNA export factors.

**Figure 1:**
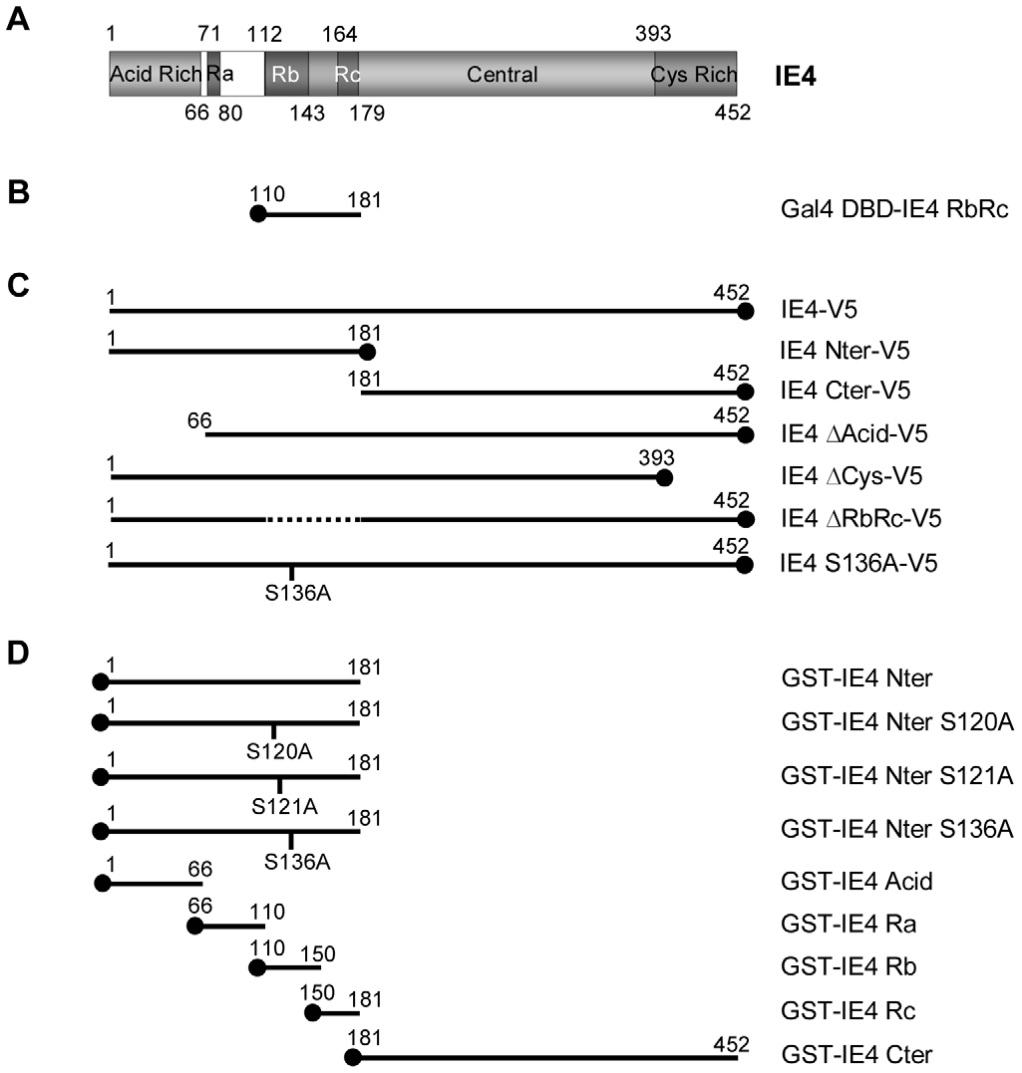
Construction of various IE4 deletants and mutants. (A) Schematic representation of the 452-amino acid coding region of IE4. The N-terminal acid-rich region (Acid Rich), the three arginine-rich regions (Ra, Rb and Rc), the central part (Central) and the C-terminal cysteine-rich domain (Cys Rich) are illustrated. (B) The RbRc yeast two-hybrid bait, (C) the multiple carboxy V5 epitope-tagged constructs as well as (D) the different amino GST fusion proteins are depicted.

Varicella-Zoster virus (VZV) is another alpha-herpesvirus encoding the IE4 protein which is homologous to the proteins described above. IE4 is rapidly produced during the first stages of infection, suggesting that it functions as an important regulator of VZV and/or cellular genes expression. The construction of IE4 knockout virus has shown that IE4 is essential for infection and has an important role in latency establishment [15], [16], [17]. However, the molecular mechanisms supported by this protein are not yet fully characterized. Based on its amino acid sequence, IE4 can be divided into four different regions (Figure 1A): (i) an acidic region located at the amino-terminal part of the protein; (ii) an arginine-rich region, also located near the N-terminus, divided into three domains called Ra, Rb and Rc; (iii) a central region; and (iv) a cystein-rich region at the C-terminus. Even if several domains seem to be multifunctional, general tendencies have been highlighted [18]: the arginine-rich domains Rb and Rc were demonstrated to be important for transactivation properties and protein-protein interactions, a nuclear localization signal (NLS) was identified within the Rb domain, and the carboxy-terminal region was also shown to be crucial for the dimerization and the cytoplasmic distribution of the protein. Although IE4 has been so far described as a transcriptional activator [19], [20], [21], its proper localization seems to be crucial for its regulatory properties, and it could be hypothesized that IE4 might exert other functions through post-transcriptional mechanisms [22], mediated by its shuttling between the cytoplasm and the nucleus.

In this report, we showed that IE4 interacts with three shuttling SR proteins, namely ASF/SF2, 9G8 and SRp20, in a yeast two-hybrid screen as well as in a cellular context. We identified the SR protein interaction domain within IE4 and demonstrated that this domain is phosphorylated by the SR protein kinase SRPK1. We showed also that IE4 is found in complexes containing TAP/NXF1 and Aly/REF, and that these complexes are stabilized by RNA since the interactions are RNase-sensitive. Finally, we established the role of IE4 in mRNA export and showed that VZV viral transcripts are exported through the TAP/NXF1 pathway.

## Results

### IE4 interacts with ASF/SF2, 9G8 and SRp20

In order to clarify the molecular mechanisms involved in IE4-mediated gene regulation and to identify IE4 potential cellular partners, a HeLa cDNA library was screened by yeast two-hybrid analysis. The region encompassing the arginine-rich Rb and Rc domains was chosen for this screening (Figure 1B). These domains have been shown to be important for transactivation, protein-protein interactions and proper localization of IE4 [18] and are therefore good candidates for the identification of cellular partners. In this experiment, 70 colonies grew on histidine-lacking media and produced β-galactosidase, meeting the criteria for positive interaction. From these colonies, 38 different proteins were highlighted, and among these the predominant category corresponds to proteins involved in RNA metabolism, which strongly suggests a post-transcriptional implication of IE4 in gene regulation. Three of the identified proteins, ASF/SF2, SRp20 and 9G8, are members of the highly conserved SR protein family.

**Figure 2:**
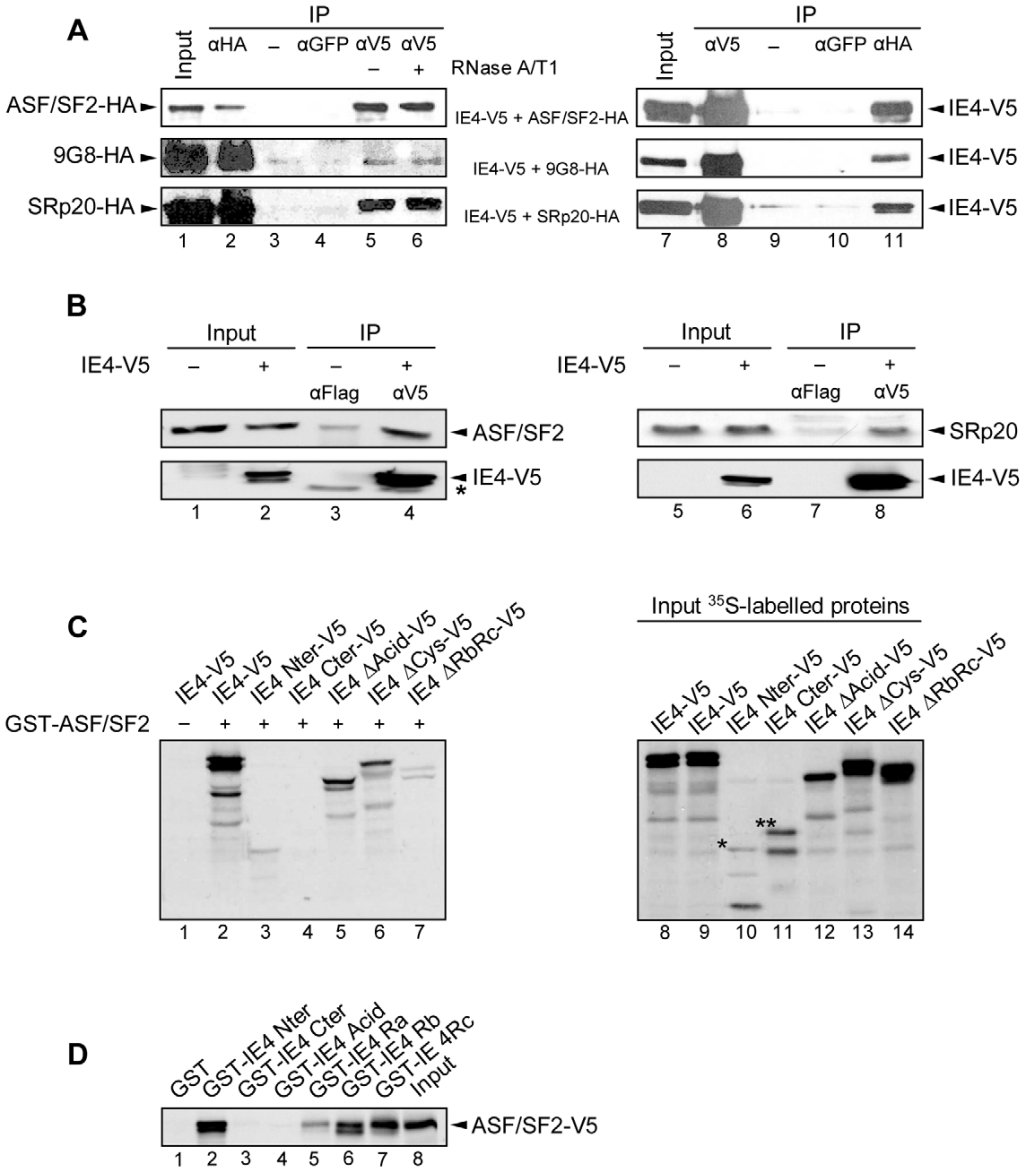
IE4 interacts with ASF/SF2, 9G8 and SRp20 through its arginine-rich Ra, Rb and Rc domains. (A) Nuclear extracts from co-transfected HeLa cells were immunoprecipitated with anti-GFP, anti-V5 or anti-HA antibodies as indicated. In Input (lanes 1 and 7), nuclear extracts were fractionated without immunoprecipitation. Nuclear extracts were treated (lane 6) or not (lane 5) with RNases A/T1 mix for 30 min at 37°C before immunoprecipitation. In lanes 3 and 9, immunoprecipitation was performed with beads without antibody as a negative control (−). The blots were probed with anti-HA (left panels) or anti-V5 (right panels) antibodies. (B) Total extracts from transfected HeLa cells were immunoprecipitated with anti-Flag or anti-V5 antibodies as indicated. In Input (lanes 1, 2, 5 and 6), total extracts were fractionated without immunoprecipitation. The blots were probed with anti-ASF/SF2 or anti-SRp20 (upper panels) and anti-V5 (lower panels) antibodies. The asterisk marks heavy chain IgG from the immunoprecipitation. (C) *In vitro* binding assay was performed by incubating GST-ASF/SF2 with *in vitro*-translated IE4-V5 or derivatives as indicated (lanes 2 to 7). Assay performed with GST alone constituted the negative control (−) (lane 1). In Input (lanes 8 to 14), [^35^S]-methionine-labelled proteins were fractionated without binding assay. (D) *In vitro*-translated ASF/SF2-V5 was incubated with GST-IE4 derivatives as indicated (lanes 2 to 7). GST alone constituted the negative control (lane 1) and, in Input, ASF/SF2-V5 was loaded without binding assay (lane 8).

To further study the interaction between IE4 and these three splicing factors, co-immunoprecipitation experiments were carried out (Figure 2A). HeLa cells were co-transfected with pcDNA3.1/V5-His-TOPO-IE4 and pCMV-HA-ASF/SF2, 9G8 or SRp20 as indicated, and either IE4 or the SR proteins were immunoprecipitated from nuclear extracts with anti-V5 or anti-HA antibodies. Immunoprecipitation efficiency was checked (Figure 2A, lanes 2 and 8) and negative controls were performed without (Figure 2A, lanes 3 and 9) or with an irrelevant antibody (Figure 2A, lanes 4 and 10). We showed that the three SR proteins are co-precipitated with IE4 (Figure 2A, lane 5) and that IE4 efficiently precipitates with the three splicing factors, no matter which partner was immunoprecipitated (Figure 2A, lane 11). To determine whether or not this interaction was mediated by RNA, we performed these co-immunoprecipitation experiments after treatment of the nuclear extracts with a mix of RNases A/T1. The three splicing factors still co-precipitate with IE4 in the presence of RNase (Figure 2A, lane 6), indicating that this interaction is not bridged by RNA.

To further confirm this interaction, co-immunoprecipitation experiments of endogenous SR proteins were also carried out (Figure 2B). HeLa cells were transfected with pcDNA3.1/V5-His-TOPO-IE4 and IE4 was immunoprecipitated from total extracts with anti-V5 antibody. Immunoprecipitation efficiency was checked (Figure 2B, lower panels, lanes 4 and 8) and negative control was performed with an irrelevant antibody (Figure 2B, lanes 3 and 7). We showed that endogenous ASF/SF2 and SRp20 are co-precipitated with IE4 (Figure 2B, upper panels, lanes 4 and 8).

### The arginine-rich region of IE4 is involved in this interaction

To determine more precisely the region of IE4 that is involved in this interaction, *in vitro* binding assays were performed by using GST fusion proteins and ^35^S-proteins that were *in vitro* translated in reticulocyte lysates. The different V5-tagged constructs (Figure 1C) were ^35^S-labelled and incubated with GST-ASF/SF2-coupled Sepharose beads (Figure 2C). Assay performed with GST alone constituted the negative control (Figure 2C, lane 1). Even if the amounts of translated ^35^S-labelled IE4 Nter-V5 (*) and Cter-V5 (**) (Figure 2C, lanes 10 and 11) are much lower than the others (Figure 2C, lanes 8 to 14), it clearly appears that IE4 Nter-V5 (Figure 2C, lane 3), as the full-length protein (Figure 2C, lane 2), is retained by the GST-ASF/SF2 fusion protein whereas IE4 Cter-V5 is not able to interact with the splicing factor (Figure 2C, lane 4), indicating that the interaction domain is exclusively located in the N-terminal part of IE4. Deletion of the acid-rich region as well as the cysteine-rich domain, involved in IE4 dimerization, does not affect this interaction as IE4 ΔAcid-V5 (Figure 2C, lane 5) and ΔCys-V5 (Figure 2C, lane 6) still bind the SR protein. Surprisingly, IE4 ΔRbRc-V5, lacking the region used as bait in our yeast two-hybrid screen, is slightly retained by the GST fusion protein (Figure 2C, lane 7), suggesting that a surrounding region could be involved in the interaction with the splicing factor. Similar results were obtained in presence of GST-9G8 and GST-SRp20-coupled Sepharose beads (data not shown).

In order to target more precisely this interaction domain, GST fusion proteins containing distinct IE4 domains (Figure 1D) were incubated with ^35^S-labelled ASF/SF2-V5 (Figure 2D). GST-IE4 Nter was shown to interact with this SR protein (Figure 2D, lane 2), while GST-IE4 Cter was not (Figure 2D, lane 3). We also showed that the acid-rich region fails to retain ASF/SF2-V5 (Figure 2D, lane 4), while the Rb and Rc domains strongly interact (Figure 2D, lanes 6 and 7). Interestingly, the Ra domain is also able to weakly interact (Figure 2D, lane 5). In the same experiment, the Ra domain slightly interacts with ^35^S-labelled 9G8-V5 and SRp20-V5 (data not shown), explaining why the IE4 ΔRbRc-V5 still interacts with the three SR proteins (Figure 2C). We thus concluded that IE4 interacts with ASF/SF2, 9G8 and SRp20 through its arginine-rich Rb, Rc and, to a lesser extent, Ra domains.

### IE4 is phosphorylated *in vitro* by SRPK1

**Figure 3:**
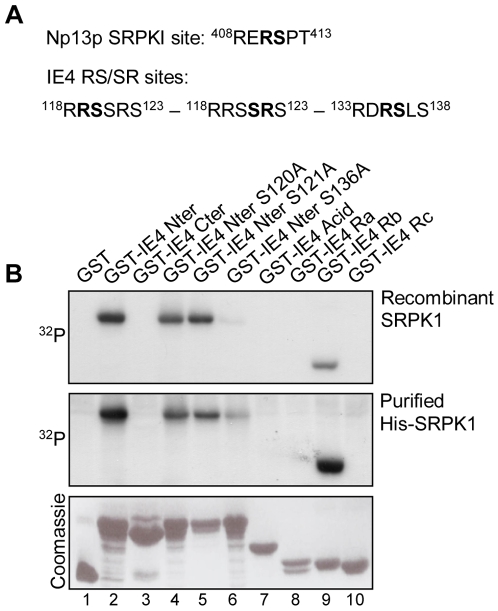
IE4 is phosphorylated *in vitro* by SRPK1. (A) The SRPK1 yeast homolog Sky1p site in Np13p as well as the putative SRPK1 sites in IE4, determined by the NetPhosK 1.0 prediction tool software, are shown. (B) *In vitro* kinase assays were performed by incubating recombinant SRPK1 (upper panel) or purified His-SRPK1 (middle panel) with GST-IE4 derivatives as substrates (lanes 2 to 10). Assay performed with GST alone constituted the negative control (lane 1). Coomassie Blue-stained gel is shown (lower panel).

SR proteins are known to be phosphorylated by SR protein kinases, such as SRPK1 [23]. Their interactions with other proteins and RNA as well as their activities are indeed precisely regulated by their phosphorylation level. In this context, the ability of IE4 to be a substrate of this kinase was investigated. *In silico* analysis of IE4 amino acid sequence, using NetPhosK 1.0 prediction tool software [24], revealed the presence in the Rb domain of three RS/SR dipeptides (Figure 3A) that resemble the SRPK1 yeast homolog Sky1p site identified in Np13p [25]. In order to determine whether IE4 was phosphorylated by SRPK1, *in vitro* kinase assays were performed (Figure 3B). The different GST fusion parts of IE4 (Figure 1D) were used as substrates and were incubated with either recombinant SRPK1 (Figure 3B, upper panel) or purified His-SRPK1 (Figure 3B, middle panel). GST alone was used as negative control (Figure 3B, lane 1). We clearly showed that the GST-IE4 Nter can be used as an SRPK1 substrate *in vitro* (Figure 3B, lane 2), whereas GST-IE4 Cter is not phosphorylated (Figure 3B, lane 3). This phosphorylation was shown to be restricted to the IE4 N-terminal Rb domain (Figure 3B, lane 9), confirming the *in silico* analysis, since GST-IE4 Acid, Ra and Rc are not phosphorylated (Figure 3B, lanes 7, 8 and 10). To target the precise phosphorylation site, the three potential phosphorylable serine residues were substituted by alanine (Figure 1D), and the mutated proteins were used in the same *in vitro* kinase assays. We showed that S120A and S121A mutations have no significant influence on the phosphorylation level (Figure 3B, lanes 4 and 5) while S136A mutation almost abolishes the IE4 phosphorylation by recombinant SRPK1 (Figure 3B, upper panel, lane 6). However, a residual phosphorylation can still be seen on the S136A mutant in presence of purified His-SRPK1 (Figure 3B, middle panel, lane 6). This could be explained by the fact that purification of His-SRPK1 from eukaryotic cells allows the presence of remaining cellular extracts, including other putative IE4 kinases which could be responsible for this weak phosphorylation. From these experiments, we can conclude that Ser-136 residue is the only amino acid phosphorylated *in vitro* by both recombinant and cellular SRPK1.

### IE4 interacts with SRPK1

**Figure 4:**
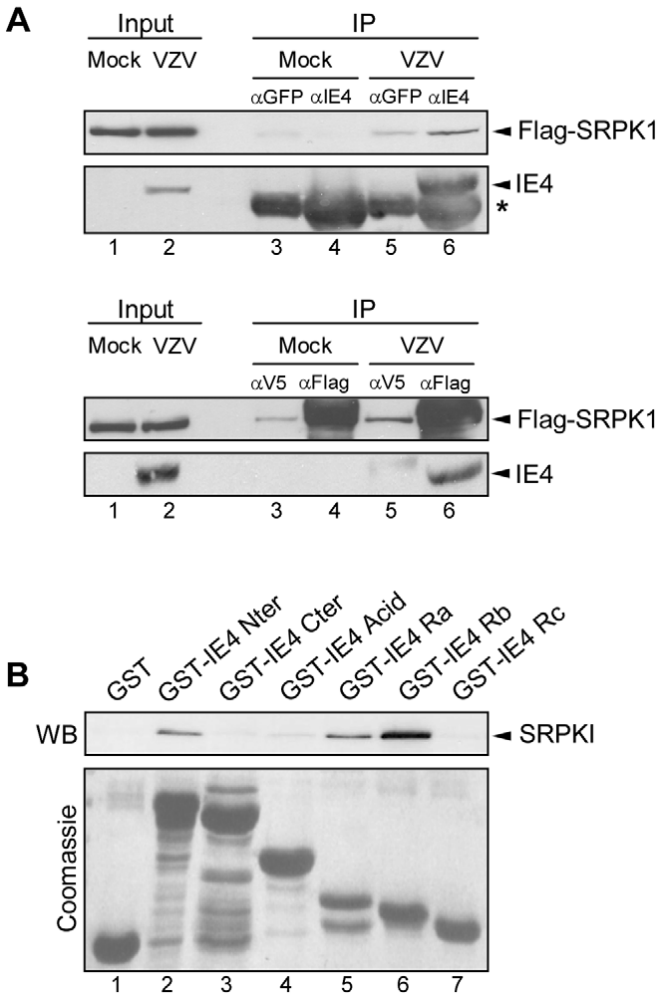
IE4 interacts with SRPK1 through its arginine-rich Ra and Rb domains. (A) Total extracts of transfected and mock- or VZV-infected MeWo cells were immunoprecipitated with anti-GFP, anti-IE4, anti-V5 or anti-Flag antibodies as indicated (lanes 3 to 6). In Input (lanes 1 and 2), total extracts were fractionated without immunoprecipitation. The blots were probed with anti-Flag or anti-IE4 antibodies. The asterisk marks heavy chain IgG from the immunoprecipitation. (B) Binding assay was performed by incubating HEK293 cells total extracts with GST-IE4 derivatives as indicated (lanes 2 to 7). Assay performed with GST alone constituted the negative control (lane 1). The blot was probed with anti-SRPK1 antibody. Coomassie Blue-stained gel is shown below.

To address the ability of IE4 to interact with SRPK1 in the context of a VZV infection, co-immunoprecipitation experiments were carried out (Figure 4A). IE4 and SRPK1 were immunoprecipitated from total extracts of pFlag-SRPK1-transfected and mock- or VZV-infected MeWo cells with specific antibodies as indicated. Irrelevant anti-GFP (Figure 4A, upper panels, lanes 3 and 5) and anti-V5 (Figure 4A, lower panels, lanes 3 and 5) antibodies were used as negative controls. Obviously, IE4 was precipitated only in VZV-infected cells (Figure 4A, upper panels, lane 6 compared to 4) and Flag-SRPK1 co-precipitates with IE4 (Figure 4A, upper panels, lane 6). Reciprocally, Flag-SRPK1 is efficiently precipitated by the anti-Flag antibody (Figure 4A, lower panels, lanes 4 and 6) and IE4 co-precipitates with Flag-SRPK1 in VZV-infected cells (Figure 4A, lower panels, lane 6).

To map the region of IE4 required for this interaction with the kinase SRPK1, binding assays were performed by using the different GST-IE4 fusion proteins (Figure 1D) incubated with HEK293 cells total extracts (Figure 4B). GST alone was used as a negative control (Figure 4B, lane 1). We showed that endogenous SRPK1 is retained by GST-IE4 Nter, Ra and Rb (Figure 4B, lanes 2, 5 and 6) while not by GST-IE4 Cter, Acid or Rc (Figure 4B, lanes 3, 4 and 7). Altogether, these results allowed us to conclude that IE4 interacts with endogenous SRPK1 through its arginine-rich Ra and Rb domains and that this interaction domain overlaps partially the SR protein binding domain.

### IE4 interacts with TAP/NXF1 and Aly/REF

**Figure 5:**
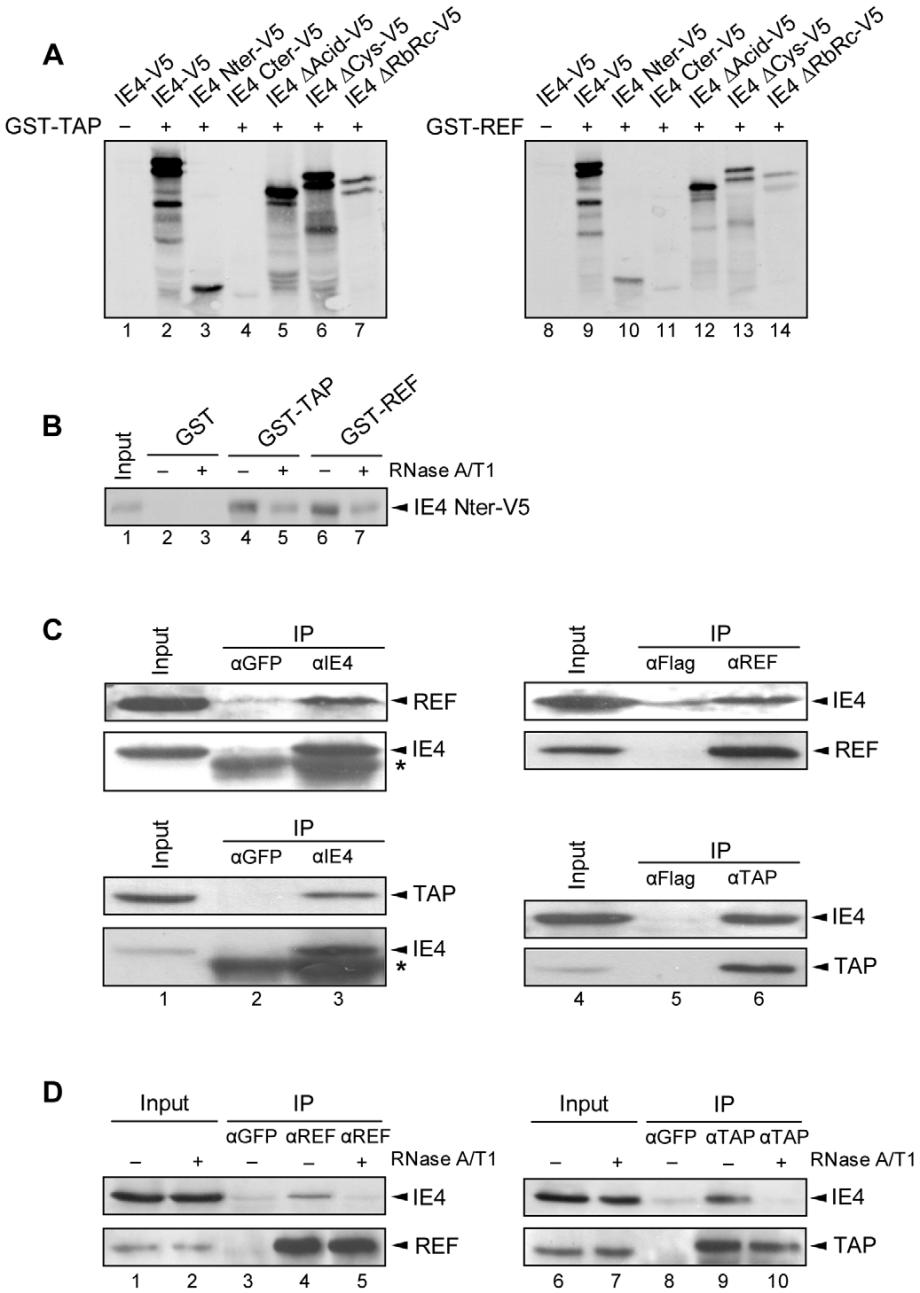
IE4 interacts with TAP/NXF1 and Aly/REF. (A) *In vitro* binding assays were performed by incubating GST-TAP (lanes 2 to 7) or GST-REF (lanes 9 to 14) with *in vitro*-translated IE4-V5 or derivatives as indicated. Assays performed with GST alone constituted the negative control (−) (lanes 1 and 8). (B) *In vitro*-translated IE4 Nter-V5 was incubated with GST-TAP (lanes 4 and 5) or GST-REF (lanes 6 and 7). GST alone constituted the negative control (lanes 2 and 3). Complexes were treated (lanes 3, 5 and 7) or not (lanes 2, 4 and 6) with RNases A/T1 mix for 30 min at 37°C before SDS-PAGE. In Input (lane 1), IE4 Nter-V5 was loaded without binding assay. (C) Total extracts of VZV-infected MeWo cells were immunoprecipitated with anti-GFP (lane 2), anti-IE4 (lane 3), anti-Flag (lane 5), anti-Aly/REF or anti-TAP/NXF1 (lane 6) antibodies as indicated. In Input (lanes 1 and 4), total extracts were fractionated without immunoprecipitation. The blots were probed with anti-Aly/REF, anti-TAP/NXF1 or anti-IE4 antibodies. The asterisk marks heavy chain IgG from the immunoprecipitation. (D) Total extracts of VZV-infected MeWo cells were immunoprecipitated with anti-GFP (lanes 3 and 8), anti-Aly/REF (lanes 4 and 5) or anti-TAP/NXF1 (lanes 9 and 10) antibodies as indicated. In Input (lanes 1, 2, 6 and 7), total extracts were fractionated without immunoprecipitation. Total extracts were treated (lanes 5 and 10) or not (lanes 3, 4, 8 and 9) with RNases A/T1 mix for 30 min at 37°C before immunoprecipitation. The blots were probed with anti-IE4, anti-Aly/REF or anti-TAP/NXF1 antibodies.

Shuttling SR proteins have been proposed to be export adaptors since, like the adaptor Aly/REF, they were shown to directly interact with TAP/NXF1 and to recruit this export receptor to bound mRNAs [11], [12]. To determine whether IE4 could be part of such export-competent mRNPs, interactions between IE4 and TAP/NXF1 or Aly/REF were first analyzed by *in vitro* binding assays. The ^35^S-labelled V5-tagged IE4 constructs (Figure 1C) were incubated with GST-TAP- or GST-REF-coupled Sepharose beads (Figure 5A). We showed that IE4-V5 is retained by both GST-TAP and GST-REF fusion proteins (Figure 5A, lanes 2 and 9). It appears also clearly that the pattern of interaction is very similar to that seen with the SR proteins (Figure 2C), since the interaction occurs through the N-terminal part of IE4 (Figure 5A, lanes 3 and 10) and is strongly reduced with the IE4 ΔRbRc mutant (Figure 5A, lanes 7 and 14).

In order to define whether or not these interactions were mediated by RNA, the complexes formed in *in vitro* binding assays between IE4 Nter-V5 and the GST-TAP or GST-REF were incubated with a mix of RNases A/T1 (Figure 5B). The interactions between the IE4 N-terminus and TAP/NXF1 or Aly/REF were shown to be sensitive to the RNase treatment (Figure 5B, lanes 5 and 7), indicating that these interactions could be bridged by RNA.

To further study the interaction between IE4 and these two factors in a context of a VZV infection, co-immunoprecipitation experiments were carried out (Figure 5C). Either IE4 or endogenous Aly/REF and TAP/NXF1 were immunoprecipitated from total extracts of VZV-infected MeWo cells with specific antibodies as indicated. Immunoprecipitation efficiency was checked (Figure 5C, lanes 3 and 6) and negative controls were performed with irrelevant antibodies (Figure 5C, lanes 2 and 5). We showed that both endogenous Aly/REF and TAP/NXF1 co-precipitate with IE4 (Figure 5C, lane 3) and that reciprocally, IE4 is efficiently precipitated with Aly/REF and TAP/NXF1 (Figure 5C, lane 6).

To confirm that these interactions were RNA-dependent, we performed these co-immunoprecipitation experiments after treatment of the total extracts with a mix of RNases A/T1 (Figure 5D). RNase treatment totally abolishes the interaction between IE4 and the two factors Aly/REF and TAP/NXF1 (Figure 5D, upper panels, lanes 5 and 10), confirming that these interactions are bridged by RNA.

### IE4 is able to bind RNA *in vitro*

**Figure 6:**
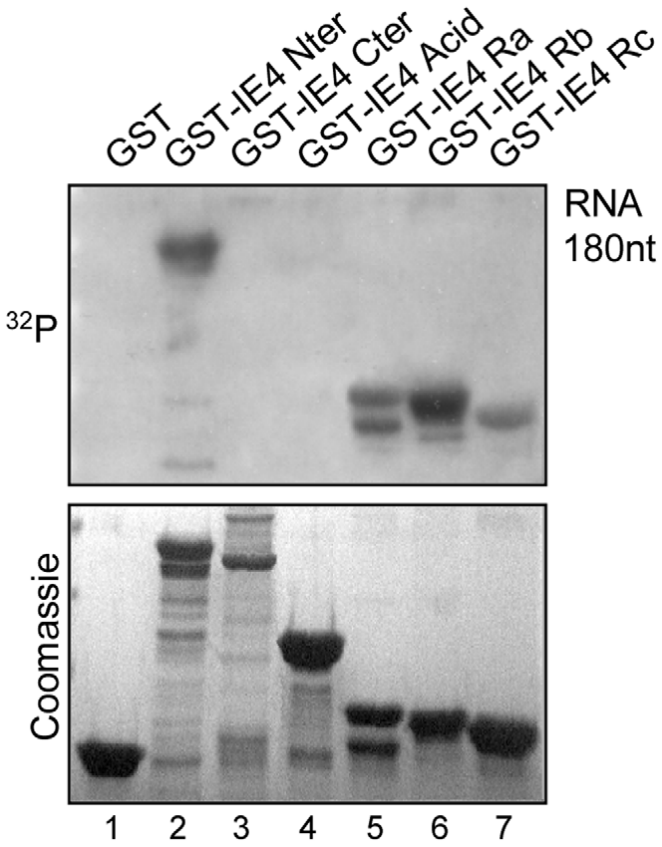
IE4 is able to bind RNA *in vitro*. Northwestern assay was performed with the GST-IE4 derivatives as indicated (lanes 2 to 7). Assay performed with GST alone constituted the negative control (lane 1). Coomassie Blue-stained gel is shown below.

As we showed that interactions between IE4 and the two factors Aly/REF and TAP/NXF1 are RNA-dependent, and to determine whether or not IE4 is able to directly bind RNA, Northwestern analysis was performed (Figure 6). Equal amounts of the different GST-IE4 fusion proteins (Figure 1D) were subjected to SDS-PAGE and transferred to a PVDF membrane. This membrane was then incubated with a ^32^P-labelled RNA probe corresponding to the human β-globin second exon RNA [26]. GST alone was used as negative control (Figure 6, lane 1). We showed that the N-terminal part of IE4 is able to bind directly to RNA *in vitro* (Figure 6, lane 2) while the C-terminal part is not (Figure 6, lane 3). More precisely, the acid-rich region fails to retain RNA (Figure 6 lane 4), while the arginine-rich Ra, Rc, and predominantly Rb domains are able to interact with the RNA probe (Figure 6, lanes 5 to 7).

### IE4 exports mRNAs in a TAP/NXF1-dependent way

**Figure 7:**
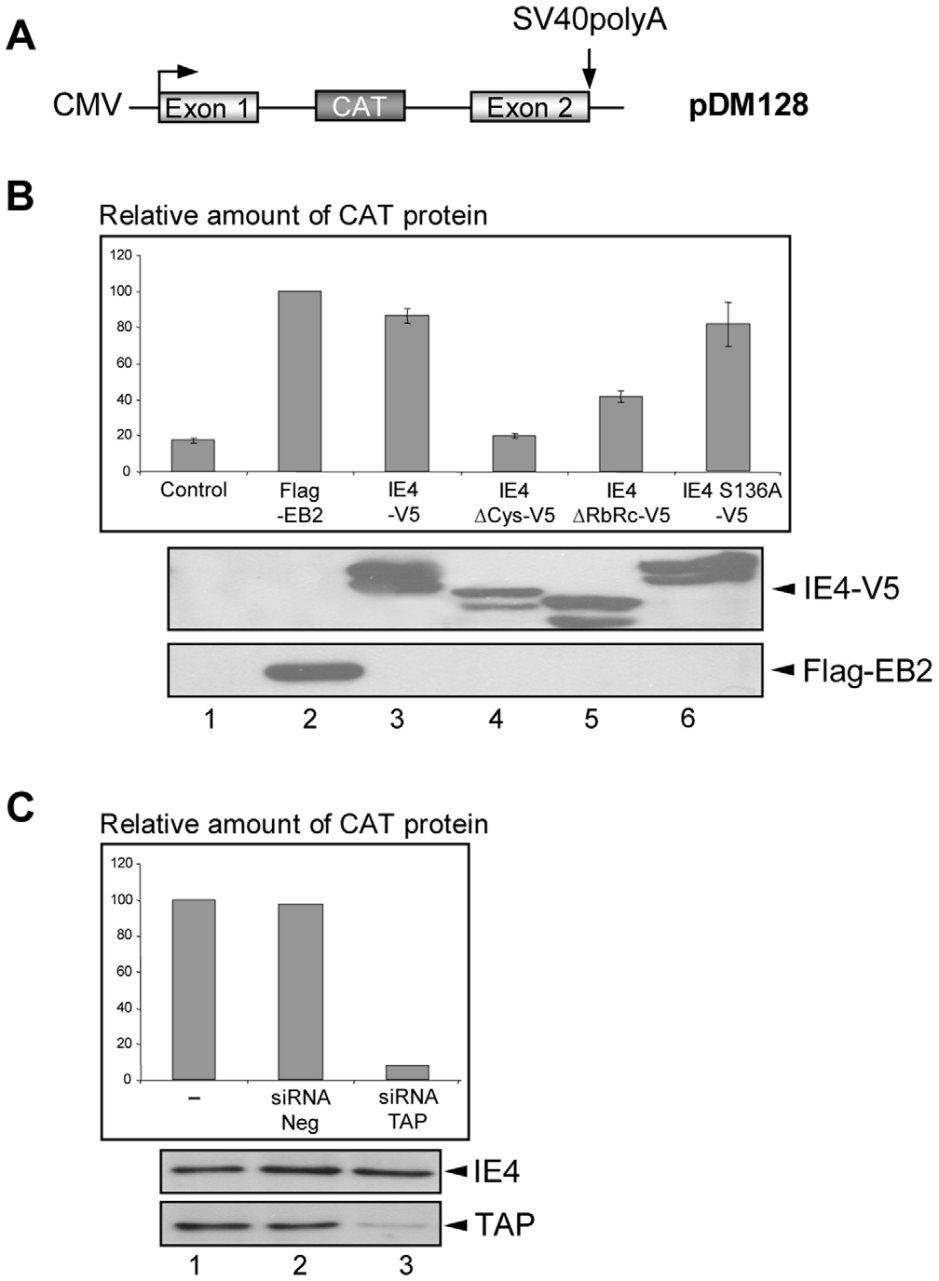
IE4 exports pDM128 mRNAs in a TAP/NXF1-dependent manner. (A) Schematic representation of the pDM128 reporter plasmid in which the CAT coding sequence is inserted into an intronic sequence (adapted from Hiriart *et al.* 2003). (B) HeLa cells were transfected with the pDM128 plasmid and expression vectors as indicated (lanes 2 to 6). Assay performed with an empty vector constituted the negative control (lane 1). CAT protein was quantified by CAT ELISA. Results are presented as a percentage of increase compared to the amount of CAT protein expressed in the presence of EB2 used as a positive control (lane 2). Expression of the different proteins was verified by Western Blotting with anti-Flag and anti-V5 antibodies. (C) HEK293 cells were transfected with the siRNA mix 24 h before CAT ELISA (lanes 2 and 3). Results are presented as a percentage of increase compared to the amount of CAT protein expressed in the presence of IE4 without any siRNA (lane 1). Expression of the different proteins was verified by Western Blotting with anti-IE4 and anti-TAP/NXF1 antibodies.

Our data showing that IE4 can interact with export adaptor SR shuttling proteins and can recruit the export factor TAP/NXF1 in a RNA-dependent manner clearly suggest a role for IE4 in the mRNA export pathway. To confirm this role, we used a functional assay extensively used to study EB2 mRNA export activity. The reporter plasmid pDM128 has been first described by Hope *et al.* [27] (Figure 7A). When transfected into HeLa cells, pDM128 expresses a two-exon, one-intron pre-mRNA which is mostly spliced, resulting in the excision of the CAT gene and very low level of CAT protein expressed (Figure 7B, lane 1). As previously described [28], EB2 expression induces the cytoplasmic accumulation of unspliced mRNAs and, therefore, efficiently increases the level of CAT protein expressed (Figure 7B, lane 2). Results are presented with a fixed value of 100 given to the amount of CAT protein detected in the presence of EB2. When a plasmid expressing IE4 is cotransfected with pDM128, the amount of expressed CAT protein reaches a level similar to what can be observed in the presence of EB2 (Figure 7B, lane 3). However, the IE4 ΔCys mutant, which was already shown to localize exclusively within the nucleus [18], appears to be incompetent in transactivating this reporter gene (Figure 7B, lane 4), suggesting that the IE4 nucleocytoplasmic shuttling capacity is required for its full activity. Moreover, the IE4 ΔRbRc mutant, although not completely inactive, fails to efficiently increase the amount of CAT protein detected (Figure 7B, lane 5), suggesting that the RbRc region, due to its interaction with RNA and the studied SR proteins, is required for the IE4 mRNA export activity. Finally, the IE4 S136A mutant, which exhibits the same subcellular localization as the wild-type protein (data not shown), appears also to be able to transactivate the reporter gene (Figure 7B, lane 6), suggesting that this phosphorylation site is not important in this mRNA export assay. Thus, the role of the phosphorylation of IE4 by SRPK1 is still to be defined.

To address the role of TAP/NXF1 in the IE4-mediated pDM128 mRNAs export, we used siRNA to knock down TAP/NXF1 expression. HEK293 cells were transfected with a mix of four targeted siRNAs for TAP/NXF1 24 h before CAT assays (Figure 7C). We showed that in cells in which TAP/NXF1 is knocked down pDM128 mRNAs are not exported anymore and CAT protein not expressed (Figure 7C, lane 3).

**Figure 8:**
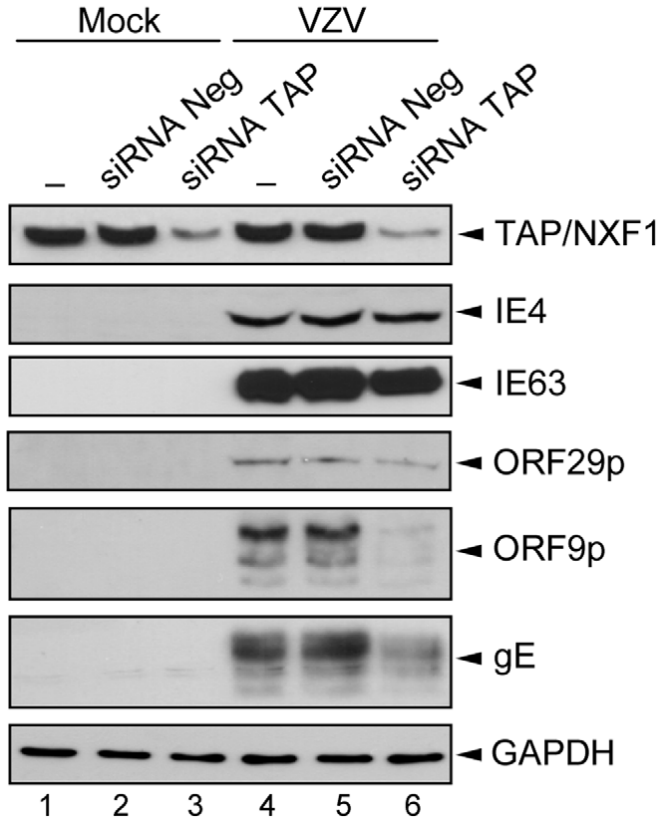
Knockdown of TAP/NXF1 alters expression of some VZV proteins. HEK293 cells were not transfected (lanes 1 and 4) or transfected with the negative siRNA pool (lanes 2 and 5) or the TAP/NXF1 siRNA pool (lanes 3 and 6) for 24 h followed by mock- (lanes 1 to 3) or VZV-infection for 36 h (lanes 4 to 6). Expression of the different proteins was verified by Western Blotting with anti-TAP/NXF1, anti-IE4, anti-IE63, anti-ORF29p, anti-ORF9p, anti-gE and anti-GAPDH (loading control) antibodies as indicated.

We have also attempted to determine the role of TAP/NXF1 on VZV gene expression (Figure 8). HEK293 cells were transfected with the siRNAs mix before being mock- (Figure 8, lanes 1 to 3) or VZV-infected during 36 h (Figure 8, lanes 4 to 6). Controls were performed without (Figure 8, lanes 1 and 4) or with a non-targeting siRNAs pool (Figure 8, lanes 2 and 5). The knockdown of the TAP/NXF1 expression was checked (Figure 8, lanes 3 and 6) and the presence of different VZV proteins from the three VZV gene classes was visualized by Western Blotting. The IE4 protein level is not affected by the TAP/NXF1 knockdown. In contrast, the ORF9p expression and, to a lesser extent, that of gE are strongly reduced in cells in which TAP/NXF1 is decreased (Figure 8, lane 6), suggesting that their transcripts require TAP/NXF1 to be efficiently exported. The expression level of IE63 and ORF29p does not seem to be substantially altered by the TAP/NXF1 knockdown, suggesting that this requirement could be a transcript- or a gene class-dependent mechanism.

## Discussion

Available data suggest that IE4 acts as an important regulator on VZV and cellular genes expression and could exert its functions at a post-transcriptional level. However, the molecular mechanisms are not yet fully characterized. In the present study, we have attempted to clarify this IE4-mediated gene regulation and identify some cellular partners of IE4. The main results obtained in this work can be summarized as follows: (i) In yeast two-hybrid, the IE4 RbRc domain interacts with cellular proteins involved in mRNA metabolism. In a cellular context, IE4 interacts with three shuttling SR proteins, namely ASF/SF2, 9G8 and SRp20, and this interaction is not bridged by RNA. (ii) IE4 strongly interacts with the main SR protein kinase, SRPK1, and is phosphorylated *in vitro* on residue Ser-136. (iii) IE4 is able to bind RNA *in vitro* and the presence of RNA stabilizes complexes containing IE4, TAP/NXF1 and Aly/REF. (iv) IE4 influences export of mRNAs through the TAP/NXF1 pathway.

SR proteins are highly conserved non-snRNP proteins whose roles in mRNA metabolism are increasingly documented. They were initially described as constitutive and alternative splicing factors but their activities in mRNA export, translation and stability are now well established. These data position the SR proteins as central components in the mRNA processing as well as highly probable targets in a viral interference on the host machinery. In retrovirus infections, such as human immunodeficiency virus type 1 (HIV-1) infection, control of alternative splicing is crucial and a number of studies showed that virion production is regulated by SR proteins, including ASF/SF2, SC35 and 9G8 [29] while SRp75 acts to upregulate HIV-1 genes expression [30]. In HSV-1 infection, ICP27 was shown to interact with essential SR splicing factors and to affect their phosphorylation impairing their ability to function in spliceosome assembly [31], [32]. Here we found a new example of a viral protein which targets the SR factors as we showed that the VZV IE4 protein interacts with ASF/SF2, 9G8 and SRp20. Interestingly, the fact that these three proteins are shuttling proteins suggests that IE4 targets their export adaptor function rather than their splicing factor activity.

The highly conserved SR protein kinase 1, SRPK1 [23], was shown to play an important role in the regulation of some fundamental cellular processes, including the organisation of components of the nucleus and mRNA processing. This may explain why SRPK1 appears to be another strategically important cellular target for several viruses. The E1̂E4 protein of human papillomavirus type 1 (HPV-1) was shown to interact and to be phosphorylated by this kinase [33]. It is still to be determined whether E1̂E4 impacts upon SR protein regulation via its interaction with SRPK1. Such behaviour has already been emphasized for ICP27 which recruits SRPK1 into the nucleus where its ability to phosphorylate SR proteins is altered [32]. This phosphorylation alteration mediates splicing inhibition which leads to host protein synthesis shut-off and promotes export and expression of intronless viral transcripts. Collectively, these findings demonstrate that viruses have adopted mechanisms to act upon SR proteins as a mean of modifying host gene expression and affecting viral growth efficiency. Here, although we identified SRPK1 as a novel cellular partner of VZV IE4, neither nuclear relocalization of the kinase nor alteration of the SR protein phosphorylation were highlighted (data not shown). Instead, we observed that IE4 interacts with SRPK1 in the cytoplasm (data not shown). Moreover, the phosphorylation mutant appeared to still be capable to export pDM128 mRNAs, suggesting that the phosphorylation by SRPK1 is not involved in the exit of IE4 from the nucleus. Therefore, it could be suggested that IE4 phosphorylation, like the rephosphorylation of shuttling SR proteins in the cytoplasm, triggers its disassembly from the exported mRNA cargo and complexes containing TAP/NXF1 and contributes to its reimport into the nucleus [34], dynamic phenomenon that we were not able to follow with the techniques that we used.

A characteristic of all known viral mRNA export factors is the presence of an RNA-binding domain. Indeed, the three IE4 homologous proteins ICP27, UL69 and EB2 were shown to possess an RNA-binding domain, which is crucial for both *in vitro* and *in vivo* RNA interaction. ICP27 was shown to require an arginine-glycine-rich region that constitutes an RGG-box RNA-binding domain [35], [36], [37] whereas EB2 and UL69 bind RNA through arginine-rich motifs which resemble other ARM motifs conserved in many RNA-binding proteins [26], [38]. Here, we showed that the arginine-rich domains of IE4 are also able to bind RNA *in vitro*, consolidating the role of IE4 as viral mRNA export factor.

TAP/NXF1 is the most thoroughly characterized mRNA export receptor, which interacts directly with the nuclear pore complex [1]. TAP/NXF1-mediated cellular mRNA export requires various adaptor proteins that associate with TAP/NXF1 and mRNP cargoes [2]. The most characterized proteins are members of the highly conserved Aly/REF family, whose recruitment to mRNAs depends on the essential mRNA export factor UAP56 [3]. Moreover, shuttling SR proteins have been recently proposed to be a second class of adaptors, as ASF/SF2 and 9G8 were shown to associate with TAP/NXF1 [11], [39]. These proteins involved in mRNA export are already known to be targeted by viral proteins in order to favour viral transcripts transport. ICP27 and EB2 were shown to bind Aly/REF in an RNase sensitive manner suggesting that RNA may be a stabilizing component of the complex [28]. In contrast, UL69 was demonstrated to bind UAP56 in absence of RNA as bridging factor [38], [40]. In addition to Aly/REF binding, ICP27 and EB2 were also reported to interact with TAP/NXF1 either directly or via Aly/REF [28], [41], which has not yet been shown for UL69. Here, we highlighted a new example of a viral protein that recruits TAP/NXF1 and Aly/REF and we showed that the interaction between IE4 and these proteins is not a direct one but rather bridged by RNA.

Several studies have shown that the herpesviral mRNA export factors described above are essential for efficient replication of the respective herpesviruses and their mRNA export activity appears to be largely involved. Indeed, the Aly/REF-binding motif of EB2 was demonstrated to be essential both for its mRNA export function and for production of infectious virions [28]. ICP27 was shown to recruit Aly/REF to viral replication compartments [41] and to access the TAP/NXF1 pathway to mediate viral mRNAs export during HSV-1 infection [42]. The KHSV ORF57 represents another example of viral mRNA export factor and its interaction with the TREX complex is required for KHSV intronless mRNAs nuclear export and viral replication [43]. In our study, we observed that, like EB2, IE4 is able to export the reporter pDM128 mRNAs. Moreover, we showed that the nucleocytoplasmic shuttling activity of the protein as well as its interaction with shuttling SR proteins and RNA are essential to mediate this nuclear mRNA export. Interestingly, the knockdown of TAP/NXF1 prevents IE4-mediated export of the reporter mRNAs, suggesting that IE4 accesses the major cellular mRNA export pathway to carry its target mRNAs to the cytoplasm. To determine the role of this mRNA export receptor during a VZV infection, we also knocked down TAP/NXF1 before infecting cells and our results indicate that TAP/NXF1 is required for the correct expression of some VZV genes suggesting a role in the export of the corresponding mRNAs. Since this observation could not be generalized to all VZV genes, we suggested that this TAP/NXF1 requirement could be a transcript- or a gene class-dependent mechanism but this specificity needs to be further explored.

**Figure 9:**
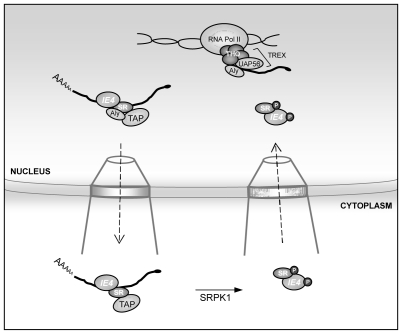
IE4-mediated mRNA export model. In this study, Varicella-Zoster virus (VZV) IE4 protein was shown to interact in the nucleus with shuttling SR proteins. These SR proteins have been previously shown to mediate interaction between intronless mRNAs and TAP/NXF1. Moreover, IE4 could be co-transcriptionally recruited to nascent mRNAs by interaction with THO proteins of the TREX complex. In addition, we have shown that IE4 is present in complexes containing TAP/NXF1 as well as Aly/REF and that these complexes are stabilized by the presence of RNA. The nucleocytoplasmic capacity of IE4, as well as its interaction with RNA and the SR proteins, have been shown to be required for its mRNA export activity. Finally, we have shown that IE4 is phosphorylated by the main SR protein kinase SRPK1, and this IE4 phosphorylation in the cytoplasm could trigger its disassembly from the exported mRNA cargo and contributes to its reimport, like it has been previously shown for the shuttling SR proteins.

All these data suggest a general mechanism for the herpesviral mRNA export factors which bind to specific viral mRNAs and recruit these transcripts to the cellular mRNA export TAP/NXF1 pathway through interaction with one of its adaptors such as Aly/REF, UAP56 or the shuttling SR proteins. The IE4-mediated mRNA export model is summarized in Figure 9. Interestingly, in our yeast two-hybrid screen, the THO complex 2 protein was also highlighted. THO proteins are known to associate with UAP56 and Aly/REF to form the elongation TREX complex. Although this interaction between IE4 and THO2 is still to be investigated, IE4 could be co-transcriptionally recruited to mRNAs and thus could couple viral transcription with nuclear export of viral mRNAs.

## Materials and Methods

### Cells

HeLa cells, a human epithelial cell line from a fatal cervical carcinoma transformed by human papillomavirus 18, and MeWo cells, a continuous human melanoma cell line, were grown in Eagle's Minimum Essential Medium (EMEM) (BioWhittaker) supplemented with 10% foetal bovine serum (FBS) (BioWhittaker) and 1% L-glutamine (BioWhittaker). HEK293 cells, a human embryonic kidney cell line, were grown in Dulbecco's Modification of Eagle's Medium (DMEM) (BioWhittaker) supplemented with 10% foetal bovine serum, 1% L-glutamine and 1% Non-Essential Amino Acid (BioWhittaker).

### Plasmids

The pcDNA3.1^−^-IE4 plasmid was described previously [18]. For the V5 epitope-tagged constructs (Figure 1C), the different IE4 gene portions were amplified by PCR from the pcDNA3.1^−^-IE4 and cloned by *TA cloning* in the pcDNA3.1/V5-His-TOPO (Invitrogen) according to the manufacturer's instructions. For the GST fusion derivatives (Figure 1D), the different portions of the IE4 gene were amplified by PCR from pcDNA3.1^−^-IE4 and cloned in the pGEX-5x (Amersham Biosciences). Mutations were introduced into the IE4 gene by PCR using the QuickChange site-directed mutagenesis kit system (Stratagene). In pGBKT7-IE4 RbRc (Figure 1A), *BamHI*-*EcoRI* IE4 RbRc sequence was inserted in frame with the Gal4 DNA-binding domain into the pGBKT7 (BD Biosciences). ASF/SF2, 9G8 and SRp20 coding sequences were amplified by PCR from the yeast-purified corresponding pGADT7 vectors (BD Clontech). These genes were cloned in the pcDNA3.1/V5-His-TOPO (Invitrogen), pCMV-HA (BD Clontech) or pGEX-5x (Amersham Biosciences) vectors in order to produce carboxy V5- and amino HA-epitope tagged constructs or GST fusion proteins respectively. All our constructions were subsequently verified by sequencing. The GST-REF and GST-TAP constructs have been described elsewhere [44] and were a generous gift from Dr. E. Izaurralde. The pCiF.EB2, encoding Flag-tagged EB2, was described previously [45]. The pRSETb-SRPK1, encoding His-tagged SRPK1, and pFlag-SRPKI plasmids were kindly provided by Dr. R. Sandri-Goldin. The reporter plasmid pDM128 has been described elsewhere [27] and was a generous gift from Dr. B. R. Cullen.

### Yeast Two-Hybrid Assay

Plasmid pGBKT7-IE4 RbRc was transformed into a mating suitable AH109 *Saccharomyces cerevisiae* strain. HeLa cDNA library, fused to the Gal4 activation domain into the pGADT7, was provided into Y187 *Saccharomyces cerevisiae* strain (BD Clontech). After mating of the two strains, co-transformants were plated on media lacking tryptophan, leucine and histidine, and then were screened for β-galactosidase production. Blue colonies, scored as positive interactions, were purified, amplified by PCR and sequenced.

### Transient Transfection and Infection

HeLa cells were transfected for 24 h before immunoprecipitation analysis, and for 48 h before CAT assay and His-SRPK1 purification. MeWo cells were transfected for 8 h before VZV infection, followed by 24 h post-infection immunoprecipitation analysis. The VZV NIK strain (a kind gift from Dr. A.F. Nikkels) was used to infect MeWo and HEK293 cells. All plasmid transfections were performed using JetPEI™ transfectant reagent (Polyplus Transfection) according to the manufacturer's instructions.

### Nuclear and Total Protein Extraction

HeLa nuclear extracts were obtained by successive incubation in hypotonic (10 mM Hepes-KOH, pH 7.9, 2 mM MgCl_2_, 0.1 mM EDTA, 10 mM KCl, 0.5% NP-40) and hypertonic buffer (50 mM Hepes-KOH, pH 7.9, 2 mM MgCl_2_, 0.1 mM EDTA, 50 mM KCl, 400 mM NaCl, 10% glycerol). HeLa and MeWo total extracts were obtained by cell lysis in immunoprecipitation buffer (20 mM Tris-HCl, pH 7.5, 150 mM NaCl, 1 mM EDTA, 1 mM EGTA, 1% NP-40, 10% glycerol) or in lysis buffer (50 mM Hepes-KOH, pH 8, 0.3 M NaCl, 1% Triton X-100). HEK293 total extracts were obtained by cell lysis in lysis buffer (25 mM Hepes-KOH, pH 7.5, 150 mM NaCl, 0.5% Triton X-100) or in lysis buffer B (30 mM Tris-HCl, pH 7, 250 mM NaCl, 1 mM EDTA, 0.1% Triton X-100, 5% glycerol) for *in vitro* binding assay. Mixture of phosphatase inhibitors (1 mM Na_3_VO_4_, 1 mM phenylmethylsulfonyl fluoride, 10 mM NaF and 25 mM β-glycerophosphate) and *Complete* protease inhibitors (Roche) were extemporaneously added in all buffers. Protein concentration was determined with a Bio-Rad protein assay.

### Immunoprecipitation

For transfected SR proteins immunoprecipitation, protein A-agarose beads (Pierce) were saturated in SR wash buffer (50 mM Tris-HCl, pH 7.4, 100 mM KCl, 0.1% NP-40) for 2 h at 4°C with anti-HA (Roche), anti-V5 (Invitrogen) or anti-GFP (SantaCruz) antibodies as indicated. HeLa nuclear extracts, treated or not for 30 min at 37°C with RNases A/T1 mix (Fermentas), were incubated with antibody-coupled beads for 4 h at 4°C in SR immunoprecipitation buffer (50 mM Tris-HCl, pH 7.4, 100 mM KCl, 5 mM MgCl_2_, 0.1% NP-40, 10% glycerol). Beads were washed in SR wash buffer and bound proteins were eluted by boiling for 2 min in SDS sample buffer. For endogenous SR proteins immunoprecipitation, protein A-agarose beads (Pierce) were saturated in immunoprecipitation buffer (see above) for 2 h at 4°C with anti-Flag (Sigma) or anti-V5 (Invitrogen) antibodies as indicated. HeLa total extracts were incubated with antibody-coupled beads overnight at 4°C in immunoprecipitation buffer. Beads were washed in immunoprecipitation buffer and bound proteins were eluted by boiling for 2 min in SDS sample buffer. For TAP/NXF1 and Aly/REF immunoprecipitation, MeWo total extracts, treated or not for 45 min at 37°C with RNases A/T1 mix (Fermentas), were precleared for 1 h at 4°C with normal rabbit or mouse IgG (SantaCruz). Extracts were then incubated for 2 h at 4°C with anti-IE4 [46], anti-TAP/NXF1 (Abcam), anti-Aly/REF (Abcam), anti-GFP (SantaCruz) or anti-Flag (Sigma) antibodies as indicated. Protein A-sepharose beads (GE Healthcare) were added for 2 h at 4°C. The precipitated were washed with lysis buffer and bound proteins were eluted by boiling for 2 min in SDS sample buffer. For SRPK1 immunoprecipitation, protein A-agarose beads (Pierce) were saturated in immunoprecipitation buffer (see above) for 2 h at 4°C with anti-IE4 [46], anti-Flag (Sigma), anti-V5 (Invitrogen) or anti-GFP (SantaCruz) antibodies as indicated. MeWo total extracts were incubated with antibody-coupled beads overnight at 4°C in immunoprecipitation buffer. Beads were washed in immunoprecipitation buffer and bound proteins were eluted by boiling for 2 min in SDS sample buffer.

### Recombinant Proteins

The various pGEX-5x-constructs were transformed in *Escherichia coli* BL21 strain and GST fusion proteins were expressed following classical induction with 0.1 mM isopropyl-1-thio-β-D-galactopyranoside (IPTG) for 4 h at 37°C. Lysates were prepared using MTPBS (150 mM NaCl, 16 mM Na_2_HPO_4_, 4 mM NaH_2_PO_4_, 100 mM EDTA, 1% Triton X-100, 1 mM PMSF, pH 7.3). Following four cycles of sonication, bacterial debris were removed by centrifugation. Proteins were then purified on glutathione-Sepharose 4B affinity beads (Amersham Biosciences), followed by extensive washing in MTPBS. SRPK1 active kinase was purified from pRSETb-SRPK1 transfected HeLa cells. ProBond Purification System (Invitrogen), for purification of polyhistidine-containing recombinant proteins, was used according to the manufacturer's instructions. Purification was performed under native conditions in order to preserve protein activity. Protein concentration was determined with a Bio-Rad protein assay.

### *In Vitro* Transcription/Translation

Proteins were expressed from pcDNA3.1/V5-His-TOPO, which contains a T7 promoter, and labelled with [^35^S]-methionine (Perkin Elmer) using the *in vitro* TNT-T7-coupled reticulocyte lysate system (Promega) according to the manufacturer's instructions.

### *In Vitro* Binding Assays

In radioactive binding assays, ^35^S-labelled proteins were incubated with GST fusion protein-coupled Sepharose beads in binding buffer (10 mM Tris-HCl, pH 8, 150 mM NaCl, 0.1% NP-40, 1 mM PMSF). Assay performed with GST alone constituted the negative control. Binding reactions were allowed to take place overnight at 4°C. Beads were washed in binding buffer. Bound proteins were eluted by boiling for 2 min in SDS sample buffer, followed by loading on 10% SDS-PAGE. Gels were subsequently transferred and autoradiographed. In non-radioactive binding assays, GST fusion protein-coupled Sepharose beads were incubated with HEK293 total extracts in MTPBS. Assay performed with GST alone constituted the negative control. Binding reactions were allowed to take place overnight at 4°C. Beads were extensively washed in MTPBS and bound proteins were eluted by boiling for 2 min in SDS sample buffer.

### *In Vitro* Kinase Assays

Equal amounts of GST fusion protein-coupled Sepharose beads were incubated with 2 units of recombinant SRPK1 (Upstate Cell Signalling) or 10 µg of purified His-SRPK1 in kinase assay buffer (50 mM Tris-HCl, pH 7.4, 10 mM MgCl_2_, 2 µM ATP) containing 5 µCi of [γ-^32^P]-ATP (Perkin Elmer). Assay performed with GST alone constituted the negative control. Kinase reactions were allowed to take place for 30 min at 30°C. Beads were washed once in MTBPS. Bound proteins were eluted by boiling for 2 min in SDS sample buffer, followed by loading on 10% SDS-PAGE. Gels were subsequently transferred and autoradiographed.

### Western Blotting

Eluted proteins were subjected to SDS-polyacrylamide gel electrophoresis (PAGE) and Western Blot analysis was used to detect the co-immunoprecipitated proteins. Endogenous ASF/SF2 and SRp20 were detected with anti-SF2/ASF (SantaCruz) and anti-SR protein (Zymed). Endogenous SRPK1 kinase was detected with anti-SRPK1 (BD Transduction). Endogenous TAP/NXF1 and Aly/REF were detected with anti-TAP/NXF1 and anti-Aly/REF (Abcam). Endogenous GAPDH was detected with anti-GAPDH (Ambion). Anti-IE4 [46] and anti-IE63 [47] have been previously described. Anti-ORF29p has been described elsewhere [48] and was a generous gift from Dr. Cohrs. Anti-gE (VL8) and anti-ORF9p were produced in our laboratory.

### Northwestern Blotting

Northwestern blots were performed essentially as described previously [49]. Briefly, GST fusion proteins were subjected to SDS-PAGE, transferred to a PVDF membrane and then denatured and renatured. The membrane was successively incubated with a ^32^P-labelled 180-nt RNA probe corresponding to the human β-globin second exon and then autoradiographed.

### CAT Assays

The reporter plasmid pDM128 has been described elsewhere [27]. CAT protein expression was evaluated using CAT enzyme-linked immunosorbent assay (ELISA) kit (Roche Applied Sciences) according to the manufacturer's instructions. Assay performed with an empty vector constituted the negative control.

### siRNA transfection

ON-TARGETplus SMARTpool of siRNAs directed against TAP/NXF1 (013680) and ON-TARGETplus Non-targeting pool (001810) were synthesized by Dharmacon Inc. Transfections were performed with 100 pmol of the siRNA pools into HEK293 cells using ProFection Mammalian Transfection System - Calcium Phosphate (Promega) according to the manufacturer's protocol. After 24 h, cells were transfected for CAT assays or VZV-infected during 36 h for Western Blot analysis. TAP/NXF1 knockdown was confirmed at both protein level by Western Blot analysis and RNA level by qRT-PCR (data not shown).
